# Telomerase promoter mutations in human immunodeficiency virus-related conjunctiva neoplasia

**DOI:** 10.1186/s12967-018-1456-0

**Published:** 2018-03-21

**Authors:** Noemy Starita, Luigi Buonaguro, Franco M. Buonaguro, Maria Lina Tornesello

**Affiliations:** Molecular Biology and Viral Oncology Unit, Istituto Nazionale Tumori IRCCS “Fondazione G. Pascale”, via Mariano Semmola, 80131 Naples, Italy

**Keywords:** TERT promoter, Mutations, Conjunctiva neoplasia, Kaposi sarcoma, HIV, Africa

## Abstract

**Background:**

Squamous cell carcinoma (SCC) of the conjunctiva is a common cancer in Africa mainly associated with solar ultraviolet (UV) exposure and human immunodeficiency virus (HIV) infection. We analyzed the role of HIV on the occurrence of telomerase reverse transcriptase (TERT) promoter mutations among a cohort of conjunctiva neoplasia Ugandan patients.

**Methods:**

Telomerase reverse transcriptase promoter mutations were searched in 72 conjunctiva neoplasia cases, comprising SCC and intraepithelial neoplasia grade 1–3 (CIN1–3), as well as in 53 conjunctiva normal tissues and in 24 HIV-related Kaposi sarcoma.

**Results:**

The average prevalence of TERT promoter mutations in conjunctiva neoplasia was 31.9%. The mutation rates were significantly higher in HIV-positive (31.8% of CIN1 and CIN2, 46.2% of CIN3 and SCC,) than HIV-negative patients (22.2% of CIN1 and CIN2, 13.3% of CIN3 and SCC). Such mutations were rarely identified among HIV-positive conjunctiva controls (3.6%) and never in Kaposi sarcoma lesions. The most frequent variations were the hot spots − 124G>A and − 146G>A and tandem transitions − 124_125GG>AA and − 138_139GG>AA.

**Conclusions:**

Telomerase reverse transcriptase promoter mutations are early events in conjunctival neoplasia and could be used for timely diagnosis of conjunctiva tumours. The high frequency of UV-signatures in HIV-positive conjunctiva lesions suggests an additive effect of the virus to UV-related mutagenesis.

## Background

Squamous cell carcinoma (SCC) of the conjunctiva is a relatively common tumour in subjects infected with the human immunodeficiency virus (HIV) living in the United States or in tropical regions of Africa [1–6]. In the United States, during the period 1996–2012, the standardised incidence ratio (SIR) for SCC of conjunctiva was 5.56 (CI 95%, 3.44–8.50) among HIV positive people versus the US general population [3]. In sub-Saharan African countries a strong association between conjunctiva SCC and HIV infection was reported since the 1980s. Indeed, the incidence of conjunctiva SCC increased more than tenfold between 1960–1971 and 1995–1997 in Kampala (Uganda) and near tenfold during the period 1991–2004 in Harare, Zimbabwe [7, 8]. More recently, a cross sectional study performed at the Kenyatta Hospital in Kenya showed that conjunctiva SCC has been the leading non-AIDS defining malignancy during the years 2000–2011 among HIV-positive patients [9].

A causal association between oncogenic viruses and conjunctival neoplasia, including human herpesvirus 8 (HHV8) as well as cutaneous and mucosal human papillomaviruses (HPVs), has been extensively searched but the results remained inconclusive [10–12].

The high prevalence of tandem CC to TT mutations identified in the TP53 gene of conjunctiva SCC DNA was suggestive of the important role played by UV solar radiation in the pathogenesis of such tumour [13, 14]. In addition, UV-related mutations along with hot spot mutations have also been identified in the promoter region of telomerase (TERT) gene in sun-exposed tumours such as melanoma, basal cell carcinoma, non melanoma skin cancer and conjunctiva SCC [15–18]. Both hot spot and UV-related mutations in TERT promoter region act as oncogenic driver events by creating binding sites for the E-twenty-six (ETS) transcription factors which generally cause a two to fourfold increase in the expression levels of TERT gene [19]. In some tumour types, including hepatocellular carcinoma and follicular thyroid adenoma, TERT promoter mutations are early events in the neoplastic process and they might be useful to monitor tumour development from dysplastic lesions [20, 21].

Very few studies have compared the mutation profile of tumours arising in HIV-positive patients versus those without HIV infection. Gleber-Netto et al. [22] analyzed the nucleotide sequence of 18 genes in HIV-related and non-HIV-related head and neck SCC and showed that among HIV-positive patients the mutations tended to be TpC>T in all mutated genes but especially in TP53. This type of nucleotide change is mainly caused by the activity of APOBEC family cytosine deaminases as host defence against viral infections which also cause nucleotide mutations in human DNA [23, 24].

In this study we have assessed the presence of TERT promoter mutations in HIV-positive and HIV-negative conjunctiva neoplasia cases to identify a possible synergistic effect of HIV on the accumulation of UV-induced mutations. Moreover, we have included in this analysis conjunctival lesions with different degree of malignancy in order to determine how early this genetic event occurs during carcinogenesis. HIV-related cutaneous Kaposi sarcoma (KS) biopsies have been also included in this study in order to verify the eventual effect of HIV status on the occurrence of TERT promoter mutations in lesions developing at body sites not exposed to sun UV radiation.

## Methods

### Patients

The cohort study comprised conjunctiva neoplasia patients surgically treated at seven countrywide eye clinics in Southern Uganda, within the Ugandan Ruharo Eye Project coordinated by Dr Waddell  KM [11]. The histological diagnosis was performed by Prof Lucas SB at the Department of Histopathology, King’s & St Thomas’ School, London, UK. The conjunctiva control tissues were obtained from healthy subjects matched to the cases by sex and age (± 10 years), which were treated for eye injuries or pterygium in the seven eye clinics. Moreover, HIV-related cutaneous African KS cases  were also included in this study [25]. All cases and controls were previously characterized in terms of histology, DNA quality, HIV serology, cutaneous and mucosal HPV as well as HHV8 DNA positivity [11, 25, 26]. The study was approved by the Institutional Scientific Board of the Istituto Nazionale Tumori “Fond Pascale”, and is in accordance with the principles of the Declaration of Helsinki.

### TERT promoter mutation analysis

Telomerase reverse transcriptase promoter region was amplified using the primer pair hTERT-F (5′-ACGAACGTGGCCAGCGGCAG-3′) and hTERT-R (5′-CTGGCGTCCCTGCACCCTGG-3′), generating a 474 bp fragment covering the rs2853669, rs34233268, rs34764648 and rs35226131 single nucleotide polymorphisms (SNPs) and the hot spot mutations within the TERT promoter region. PCR negative samples were further amplified with the primer set hTERT_short-F (5′-CAGCGCTGCCTGAAACTC-3′) and hTERT_short-R (5′-GTCCTGCCCCTTCACCTT-3′) which amplifies a sequence of 163 bp encompassing the TERT promoter hot spot sites. PCR reactions were performed in 50 μl mixture containing 300 ng of genomic DNA, 10 pmol of each primer, 1.25 Unit of Hot Master Taq DNA Polymerase (5 Prime GmbH, Hamburg, Germany) and 25 μl of PreMix J (Master Amp PCR, Epicentre). DNA was amplified in the Sure Cycler 8800 thermal cycler (Agilent Technologies) with the following steps: an initial denaturation at 94 °C for 3 min, followed by 32 cycles of annealing at 65 °C for 30 s when using hTERT-F/-R primer set or at 53 °C for 30 s when using hTERT_short-F/-R primer set, elongation at 72 °C for 1 min, denaturation at 94 °C for 30 s, and 10 min final elongation at 72 °C. All amplified DNA samples were subjected to automated bidirectional sequencing analysis at Eurofins Genomics, Munich, Germany. Nucleotide sequences were edited using the BioEdit software package (http://jwbrown.mbio.ncsu.edu/BioEdit/bioedit.html).

### Statistical analysis

The statistical analyses were performed using Graph Pad Prism Software version 6.00. Two-tailed Χ^2^ test, Χ^2^ test for trend or Fisher’s exact test were used for comparison of categorical data. Differences were considered statistically significant when *P* values were less than 0.05.

## Results

This study included a total of 72 cases of conjunctiva neoplasia, comprising 16 CIN1, 15 CIN2, 17 CIN3 and 24 SCC. Fifty-three conjunctiva non-neoplastic controls and 24 HIV-related KS lesions were also analysed in this study (Table 1). The majority of patients and controls were positive for HIV infection (66.7 and 52.8%, respectively).

**Table 1:** Distribution of known variables of conjunctival neoplasia cases and controls

	Conjunctiva neoplastic tissues	Conjunctiva control tissue^a^	*P* value
	*N *= 72 (%)	*N *= 53 (%)	
Sex			0.031
M	29 (40.3)	32 (60.4)	
F	43 (59.7)	21 (39.6)	
Age			1.000
≤ 30 years	33 (45.8)	24 (45.3)	
> 30 years	39 (54.2)	29 (54.7)	
TERT promoter mutations			0.0001
Yes	23 (31.9)	1 (1.9)	
No	49 (68.1)	52 (98.1)	
HIV serology			0.141
Positive	48 (66.7)	28 (52.8)	
Negative	24 (33.3)	25 (47.2)	

^a^The control group comprises one benign lesion (conjunctiva papilloma) from an HIV-negative subject

Overall, TERT promoter mutations were detected in 23 out of 72 (31.9%) conjunctiva neoplasia cases, in one out of 53 (1.9%) control tissues and were absent in KS lesions (Table 1). The frequency of mutations was found statistically significantly higher in the group of HIV-positive CIN3 and SCC (46.2%) compared to HIV-negative CIN3 and SCC cases (13.3%), P = 0.04, (Table 2). Similarly, a higher mutation rate, although not reaching statistical  significance, was observed among HIV-positive CIN1 and CIN2 (31.8%) compared to HIV-negative cases (22.2%). Moreover, the occurrence of TERT promoter mutation in conjunctiva neoplasia was not affected by the HPV or HHV8 infection status.

**Table 2:** Cutaneous and mucosal HPV and HHV8 infection as well as TERT mutation frequency stratified by conjunctival neoplasia grades and HIV status

	CIN3 + SCC (*HIV*+*)*	CIN3 + SCC (*HIV*−)	CIN1 + CIN2 (*HIV*+)	CIN1 + CIN2 (*HIV*−)	Control conjunctiva (*HIV*+)	Control conjunctiva (*HIV*−)
	*N *= *26* (%)	*N *= 15 (%)	*N *= 22 (%)	*N *= 9 (%)	*N *= 28 (%)	*N *= 25 (%)
HPV-Pos	4 (15.4)	0 (0.0)	7 (31.8)	2 (22.2)	0 (0.0)	1 (4.0)
HPV-Neg	22 (84.6)	15 (100.0)	15 (68.2)	7 (77.8)	28 (100.0)	24 (96.0)
HHV8-Pos	3 (11.5)	5 (33.3)	5 (22.7)	2 (22.2)	0 (0.0)	1 (4.0)
HHV8-Neg	23 (88.5)	10 (66.7)	17 (77.3)	7 (77.8)	28 (100.0)	24 (96.0)
TERT-Mut^a^	12 (46.2)	2 (13.3)	7 (31.8)	2 (22.2)	1 (3.6)	0 (0.0)
TERT-Wt	14 (56.8)	13 (86.7)	15 (68.2)	7 (77.8)	27 (96.4)	25 (100.0)

^a^CIN3 + SCC HIV-positive versus HIV-negative, P value 0.04

**Fig. 1:**
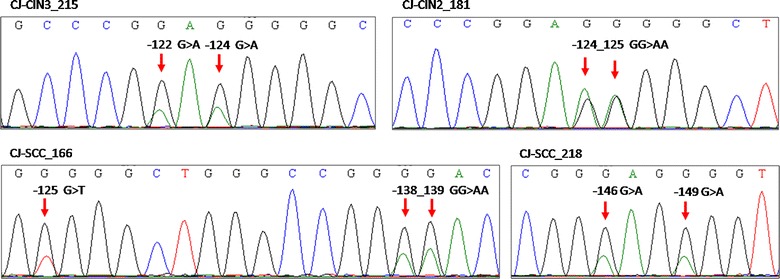
Representative sequence electropherograms of hot spot and sporadic mutations in TERT promoter region of conjunctiva neoplasia samples

The most common nucleotide changes were the hot spot mutations − 124G>A (17.4% of all mutated cases) and − 146G>A (21.7%) as well as the UV-related tandem mutations − 124_125GG>AA and − 138_139GG>AA which together added up to 43.5% of all mutated cases. The two hot spots and the UV-related tandem mutations were found mutually exclusive while sporadic changes were detected as additional variations. Particularly, two cases containing the − 124G>A transition also carried a G>A mutation at position − 101 or − 122 from the ATG TERT start site. One sample harbouring the mutation at nt − 146 also contained two additional G>A transitions at nt − 100 and − 149. Tandem mutations − 124_125GG>AA and − 138_139GG>AA were also accompanied by sporadic G>A transitions at nt − 102 in one case and at − 101 and a G>T transversion at position − 125 in another case. Most of the observed changes lead to the creation of putative transcription factor-binding sites, such as the ETS-binding motif and SPI1 or ELK1 binding sites (Table 3). All nucleotide changes were heterozygous with one affected allele (Fig. 1).

**Table 3:** Pattern of TERT promoter mutations in conjunctival neoplasia and conjunctiva control tissues

TERT mutations^a^	Effect^b^	CIN3 + SCC	CIN1 + CIN2	Control conjunctiva
		*N *= 41 (%)	*N *= 31 (%)	*N *= 53 (%)
− 101G>A	SPI1	3 (7.3)	1 (3.2)	0 (0.0)
− 124G>A^c^	ETS	2 (4.9)	2 (6.5)	0 (0.0)
− 124_125GG>AA^d^	ELK1	4 (9.8)	1 (3.2)	1 (1.9)
− 138_139GG>AA^e^	ETS	3 (7.3)	2 (6.5)	0 (0.0)
− 146G>A^f^	ETS	2 (4.9)	3 (9.7)	0 (0.0)
All mutations^g^		14 (34.1)	9 (29.0)	1 (1.9)

^a^Positions refer to the distance from the ATG start site of TERT gene

^b^Putative transcription factors binding sites identified in JASPAR database (http://jaspar.genereg.net)

^c^One sample carried an additional mutation at nt − 101G>A and another at nt − 122G>A (no effect on putative binding sites)

^d^One sample carried an additional mutation at nt − 102G>A (no effect on putative binding sites)

^e^One sample carried two additional mutations at nt − 101G>A and nt − 125G>T with no effect on putative binding sites

^f^One sample carried two additional mutations at nt − 100G>A (no effect on putative binding sites) and nt − 149G>A (creating a putative ETS binding site)

^g^Χ^2^ for trend = 16.13, P = 0.00006

Only one HIV-positive case among the conjunctiva control samples harboured a − 124_125GG>AA change suggesting that UV-related mutations may precede the development of conjunctival low grade neoplasia (Table 3). No mutations were identified in TERT promoter region of DNA extracted from HIV-negative conjunctiva controls or HIV-positive cutaneous KS samples.

Several single nucleotide polymorphisms (SNPs) are present within the TERT locus and some have been associated with the risk of cancer. In the present study, the TERT promoter region amplified with hTERT-F and hTERT-R primer set encompassed four SNPs and their allele frequencies have been evaluated in conjunctiva cases and controls. Particularly, a higher frequency of minor alleles (MAF) among cases versus controls was observed for the rs2853669 (− 245 G, MAF = 0.15 and MAF = 0.10, respectively), the rs34233268 (− 218 C, MAF = 3.3 and MAF = 1.9, respectively) and the rs35226131 (− 269 T, MAF = 3.3 and MAF = 1.9, respectively), however such differences did not reach a statistical significance. On the other hand, similar allele distribution has been observed for the rs34764648 (− 354_355, ΔCΔG) with MAF = 5.9 and MAF = 5.7 in cases and controls, respectively.

## Discussion

The solar UV radiation exposure is the main cause of the most common skin cancers, such as basal cell carcinoma, cutaneous SCC, cutaneous melanoma and other epithelial tumours such as conjunctiva neoplasia [27, 28]. The frequency of C>T or CC>TT UV-related mutations in TERT promoter region has been found similarly high in basal cell carcinoma (56%), cutaneous SCC (50%), cutaneous melanoma (up to 71%) and conjunctiva neoplasia (43.8%), [16–18, 29].

The pattern of TERT promoter mutations identified in our Ugandan conjunctiva neoplasia cohort is similar to that previously described in conjunctiva SCC of German patients as well as in melanoma and non-melanoma skin cancers [17, 18, 29, 30]. Interestingly, the occurrence of TERT promoter mutations in 37.5% of CIN1 observed in our results suggests that the UV-induced DNA damage may precede the progression of conjunctiva early lesions to high grade neoplasia.

Several studies demonstrated that HIV infection strongly increases the risk of conjunctiva neoplasia [12]. HIV-related immunosuppression has been demonstrated to play a key role in such association (Holkar et al. [31]; Grulich et al. [32]). In our cohort the frequency of UV-related TERT promoter mutations was significantly higher in HIV-positive compared to HIV-negative conjunctiva neoplasia cases suggesting a synergistic effect of the virus with UV in the accumulation of DNA damages. No biomolecular study has systematically analyzed the effect of HIV on the occurrence of UV-related mutations, however, such phenomenon is supported by epidemiologic studies showing that the incidence of basal cell carcinoma and cutaneous SCC was 2.1-fold higher and 2.6-fold higher, respectively, among HIV-positive patients compared with HIV-negative subjects [33]. In their study, the increased risk of skin cancer was correlated with lower CD4 counts in squamous cell carcinoma but not among basal cell carcinoma among HIV-positive patients suggesting that immunosuppression was only partially responsible for the increased incidence of skin cancer.

A recent report compared the pattern of mutations among HIV-related and non-HIV related head and neck SCC in genes known to be frequently mutated in such tumours and identified a different pattern of nucleotide changes in all mutated genes including TP53 [22]. Particularly, they observed an enrichment of C>T changes in the HIV-infected cases likely caused by the cytosine deamination [34]. We have observed a high rate of single C>T or tandem CC>TT changes in HIV-related conjunctiva lesions but not in HIV-related Kaposi sarcoma suggesting a synergistic effect of UV exposure and HIV infection but not a direct effect of HIV in not UV-related cancers such as Kaposi sarcoma.

The hot spot nucleotide changes − 124G>A and − 146G>A in TERT promoter have been detected at high frequency in cancers of internal organs, such as bladder cancer, hepatocellular carcinoma, thyroid cancer, and gliomas [15, 35, 36]. In contrast, tandem mutations CC>TT are related to the UV mutagenic activity and very rarely identified in tumours of internal organs [13, 29, 35, 35–39]. In our study CC>TT substitutions in TERT promoter were very frequent in accordance with their overall frequency in other UV-related tumours [16, 40].

TERT promoter mutations create de novo binding motifs for the ETS (E-twenty-six) family or TCF (ternary complex factor) subfamily of transcription factors and increase the expression of TERT by twofold to fourfold [18]. This increased telomerase expression enables tumours to maintain their telomere length and continuously proliferate without becoming apoptotic or senescent due to genetic instability [41, 42].

Several SNPs have been analyzed in conjunctiva samples, including rs2853669, rs34233268, rs34764648 and rs35226131, but no significant differences have been noted on the minor allele frequency distribution among cases and controls. However, the limited number of samples insufficient to perform a significant statistic may have hindered the possibility to associate specific SNPs with susceptibility to conjunctiva neoplasia.

## Conclusion

In conclusion, we observed that HIV infection, a major risk factor for development of conjunctiva neoplasia, significantly contributes to the accumulation of UV-related mutations in TERT promoter. Both hot spot mutations and UV-related variations are frequently identified in low grade conjunctiva lesions (CIN and CIN2) as well as in high grade lesions (CIN3) and invasive carcinoma. More studies are needed to understand the molecular mechanisms underlying this previous unknown phenomenon and to determine whether these genetic traits are useful for early detection of conjunctiva progressing lesions.

## Abbreviations

TERT: telomerase reverse transcriptase
SCC: squamous cell carcinoma
CIN: conjunctival intraepithelial neoplasia
HIV: human immunodeficiency virus
